# Notes on Genito-urinary Therapeutics

**Published:** 1883-01

**Authors:** 


### NOTES ON GENITO-URINARY THERAPEUTICS.

(*Continued from vol. 2, page 272.*)

ALUMINA. RENAL.—Pain in kidneys and loins (as from jolting over a rough road), alternating with cloudiness, as if drunk; pain is worse on stooping or turning (pain after stooping: Natr. m., Sulph.).

Feeling as if a hook were plunged into her, which makes her scream.

VESICAL.—Pressure and drawing in the region of the bladder, especially at neck of the bladder.

Feeling of weakness in bladder and genital organs.

Fears he will wet his bed (in the evening).

Violent tenesmus.

Micturition frequent at night.

Frequent micturition (urine corroding) during the menses. (Sarsap.)

*Urine voided only while straining at stool,* requires such force to pass it. (Slow emission, bladder weak, has to wait before urine will pass; must strain so to pass it that anus protrudes: Mur. ac.)

URINARY PAINS.—Sensation of heat in urethra, which passes off by patient lying still.

Severe smarting and burning, with feeling as though a few drops remained in urethra which could not be expelled. (Arg. n., Kali b., Thuja.)

Frequent smarting and corroding urine (with prolapsus uteri).

During urination, burning and desire to evacuate bowels.

Cutting in anterior part of urethra during micturition, and even after, as if urine passed over an inflamed surface.

Urethra feels hot after emission of urine, followed by sensation as of burning and tenesmus of rectum and bladder.

Increased secretion and frequent discharge of pale, watery urine.

CHARACTER OF URINE.—White, turbid urine, as if chalk had been stirred in it.

Pale urine with turbid sediment.

When standing, the urine deposits a thick white sediment.

Deep yellow urine; soon deposits a large and loose cloud.

Scanty, with red sediment (in arthritic affections).

Copious and pale (in nervous diseases).

Copious and dark.

SEXUAL.—Desire excessive.

Emissions at night, with voluptuous dreams.

Emission during the afternoon nap (Canth., Staph.). (These emissions aggravate patient's old symptoms.)

Contractive pain in right spermatic cord; the testicle of that side is drawn up at same time, and is sore and painful.

Left testicle is hard and indescribably painful to the touch. (Hard: *Acon.*, *Agn.*, Arg. n. (r.), *Arn.*, Clem., Lach., Merc., Nux v., Ph. ac., RHOD., Sab., Sil. (r.), SPONG., Strych. (l.).

Itching of the scrotum; relieved by scratching.

Tickling on genitals and thighs.

AMBRA. VESICAL.—Pain in bladder and rectum simultaneously.

Sensation as if a few drops passed out of the urethra.

Burning in the orifice of the urethra.

CHARACTER OF URINE.—The urine is dark-brown and a little turbid, even while being emitted.

Urine turbid, even while being emitted; yellow-brown color; it formed a *brown sediment* (Chin. s., Crot. t., Dig., Val.), leaving the urine above clear and yellow.

CONCOMITANT.—Frequent urging but no stool; this gives her a great deal of anxiety; during this time THE PRESENCE OF OTHER PERSONS WAS INTOLERABLE.

AMM. CARB. VESICAL.—Violent tenesmus of the bladder, with cutting.

Constant tenesmus, even at night, with diminished passage of urine, accompanied by burning.

Blood comes out of the urethra.

URINARY PAINS.—After urinating, there is a strong traction in forepart of the urethra.

Cutting, burning.

CHARACTER OF URINE.—Copious flow; increased micturition. night.

Involuntary, toward morning, during sleep.

Urine very muddy, of a peculiar smell, with very copious sediment.

Pale urine, with sandy sediment.

Increased and turbid urine.

SEXUAL.—Forcing (choking) pain in testicles and seminal chords; sensitiveness of testicles to touch, aggravated by erections.

Testicles and scrotum relaxed, necessitating a supporter.

Occasional drawings in the scrotum, which are relieved by raising it.

Erections without sexual desire, mornings.

Violent desire, with trembling of body, almost without any erection.

Seminal emissions almost every night.

Itching on glans penis, which lasted several days.

Itching of genitals, especially scrotum.

